# The Hospitals

**Published:** 1874-09

**Authors:** 


### The Hospitals.

***Cook County Hospital.*** July 10, 1874. Medical Clinic. By Dr. H. M. Lyman.

A woman with chronic diarrhœa, tongue very red, was receiving four times a day teaspoonful doses of turpentine emulsion, to which was added a little laudanum. There is one fluid drachm of turpentine to the ounce.

A patient with acute gastritis (vomiting of blood, and likewise discharging the same from the nose), due to alcoholic excesses, was receiving scruple doses of potassium bromide every two hours.

A German, of thirty-one years of age, was under treatment for the insomnia and restlessness attendant upon a drunken spree. He received the following: R. Potassii bromidi, oz. j; chloral hydratis, dr. vj; syrupi simplicis, aquæ, *aa* f. oz. ij. M. Sig. one teaspoonful every four hours.

Surgical Clinic, by Drs. Bogue and Freer.

The case of osteo-aneurism, reported in the last number of the Journal, terminated fatally July 5th. The patient had left the hospital soon after the operation, but continued to lose strength. A fungoid growth sprung up in the cavity of the wound, attended with capillary oozing and hemorrhages, which resulted at length in death.

*Pleuritic Abscess—Fracture of Right Humerus—Luxation Right Shoulder, four months' duration—Railroad Injury of the Right Foot, Syme's Operation.*

A boy, five years of age, had empyema of the right chest, which had opened externally, spontaneously. Dr. Bogue enlarged the opening and inserted a drainage tube, previously having passed a thread through the rubber tube, to prevent its slipping into the thoracic cavity.

A fracture of the right humerus, due to a fall, was treated by extension, weight, and pulley.

There was no interference in the case of a German, who had a dislocation of the right shoulder of four months' duration. Some details were given by Dr. Freer, of a list of accidents that had occurred in twenty cases, where attempts had been made to reduce this kind of injury. In India, a dislocation of this sort had been reduced after two years' displacement.

A young Swede had his foot mashed by a railroad accident. Dr. Freer performed Syme's operation, using Esmarch's bandage, and ether as an anesthetic.

In regard to Esmarch's bandage—a practical and recent application of an old discovery—Dr. Freer answered some objections to it, made by Langenbeck, as, for instance, the danger from septicæmia, where the decomposed fluids of a limb might be sent into the system by bandaging. In such case, there would be no need of bandaging below the seat of infection—begin higher up. To the second objection, that the bandage endangered the well-being of the flaps, conducing to sloughing, it was said that there would be sloughing in a certain per cent. of cases if no bandage were used. There has been no case of sloughing of flaps in the Cook County Hospital which could be attributed to the use of Esmarch's bloodless method. If there were ecchymoses along the limb (though he had seen none at this hospital), it was probably due to applying the bandage too tightly.

July 21. Medical Clinic. By Dr. LYMAN.

*Scarlatina Hemorrhagica.*

The patient, a brass-finisher, a Canadian, twenty-two years of age, thought this was his second attack of scarlet fever, and that the eruption came out on the eighth day of his sickness. The eruption was coextensive with the entire body—confluent on the face only; the limbs were lowered in temperature, and covered with large purpuric patches; the color did not disappear upon pressure. The tongue, buccal and faucial membranes were intensely red; the edge of the gum, next the teeth, was marked by a line of apparent hemorrhagic exudation.

Treatment: Potassium chlorate, compound tincture of cinchona; brandy and water; and the following: R. Potassæ

acetatis, oz. ss; tr. digitalis, f. oz. j; aquæ, f. oz. iij. M. Sig. Take one teaspoonful four times a day.

*Diabetes.*

This patient, an Irish laborer, thirty-six years of age, was receiving every six hours teaspoonful doses of turpentine emulsion, to which were added eight minims tr. opii. The citrate of iron and quinine was given three times a day. He is now troubled by a diarrhœa; tongue red and much furrowed. His urine is of the specific gravity of 1041, and he voids ten and a half quarts daily. The desire to drink is strong and constantly present. In 1866 he had an attack of sunstroke.

July 28. Dr. H. M. LYMAN.

*Scorbutus.*

At present there are four cases of land-scurvy under treatment. One patient is a Norwegian, thirty-five years of age, and a cabinet-maker. The other patients are boys, two of whom are eight years, the third nine years of age, and inmates of a school in which they state they have seldom had potatoes or onions to eat. One of the boys has lost two teeth. His gums are blue, very spongy, and crowd between the teeth. The face is a little swollen.

The Norwegian had been boarding himself, and living in a damp, dark and underground apartment, beneath a barn. He says he has had no vegetables in the last three years, and lived on salt pork the last two years. His weakness is extreme. He is almost bloodless, and very emaciated; legs painful, stiff and swollen, and covered with petechiæ and purpuric patches. He has lately lost four teeth; his breath is very fœtid; gums tumid and tender. His bowels have moved only twice in the past month. Dr. Lyman adverted to the proverb among sailors, that the sight of land would kill scorbutic sailors, and explained it as not being due to that cause, but to the over-exertion and excitement of the sailor *in trying to get up on deck* to see land, when his anæmic and asthenic condition ought to have forbidden his getting up at all; there would be danger of fatal syncope from such imprudence. Ordered nutritious and vegetable diet, acid drinks, and tr. ferri muriatis; also a permanganate of potassa gargle.

A German gardener, thirty-five years of age, had an attack of remittent fever eight years ago; more recently, an attack of intermittent fever. His present condition is that of malarial cachexia and anæmia. There was also the well-known ague cake; the spleen upon percussion and palpation was found to be a hardened mass, with a distinct indurated edge.

Ordered: R. Ferri et quiniæ citratis, gr. iij; acid arseniosi, strychniæ sulphatis, *aa* gr. $\frac{1}{30}$. M. Ft. fil. No. 1. Sig. Take one such pill three times a day, after meals. Locally, apply over the region of the spleen an ointment containing the biniodide of mercury. It will be necessary to continue the above treatment not less than two months.

Valvular disease of the heart in an Irish woman, seventy-nine years of age. If there is dilatation and thinness of the cardiac walls, with feeble action, you may exhibit tincture of digitalis; doses of twenty drops. If there is hypertrophy, give veratrum viride up to toleration; dose of three or four minims; and you can give more of it by combining with it the tr. opii, which will be an antidote to the depressing effects of veratrum viride.

---

***Rush Medical College.*** Saturday, July 18. Surgical Clinic, by Dr. Gunn.

*Abscess of the Left Side.*

Dr. Gunn brought before the class a man forty-five years of age, who had had pleuro-pneumonia with effusion upwards of two years, together with pneumo-thorax. Six weeks after the attack, two openings in the left chest formed spontaneously, and had continued to discharge pus for more than a year. The left half of the chest is the smaller, by more than two inches. These closed up, but again re-opened. The patient desired to know whether there was any more fluid in the chest, and whether there would be a third opening and discharge? He was informed that there was probably no fluid remaining.

Dr. Gunn remarked that he believed he was a pioneer of what has of late years become the practice, namely, early tapping in effusions of the chest. It is the duty of the physician to tap patients with effusion at the end of three weeks, if even it be only serum, for the possible danger to the lung by compression is very great. He did not think the danger from air in the chest was very great, and cited several cases in support of his view.

Where the aspirator is not at hand, the following is the mode of tapping: Place the patient over the edge of the bed, and with a thumb lancet make a vertical incision, one inch long, directly over the seventh rib; draw up the skin, and in the sixth intercostal space, near the seventh rib, puncture the chest wall. A valvular incision will have thus been made, into which a pledget of lint may be inserted, and secured by adhesive plaster; the skin may slide over it. The next day it may be withdrawn, to draw off more fluid. After a while the discharge will cease, and the fistulous tract close up.

---

***Mercy Hospital.*** July 20.

Your reporter desires to acknowledge the courtesies received from Dr. Haines, the House Physician.

A patient who had had a bronchitis of long standing, with emphysema, and been subjected to many forms of treatment without cure, was given, tentatively, the potassium iodide with immediate relief and benefit. Upon substituting hypodermic injections of the biniodide of mercury (gr. $\frac{1}{32}$), he relapsed, and had periosteal trouble. The iodide of potassium was resumed, forty grains daily, and to the nodes an application was ordered of: R. Acidi carbolici, f. dr. j; tr. iodinii, f. dr. vij. M.

*Acute Articular Rheumatism.*

The usual treatment is to render the urine alkaline within forty-eight hours after admission, if possible. This is done by the exhibition of half-drachm doses of the acetate or citrate of potassa, with suitable doses of wine of colchicum. Sometimes the nitrate of potassa is used. Dover's powder, to quiet pain. No topical applications. After two days, this treatment is suspended, and three-minim doses, four times a day, of the strong nitro-muriatic acid, with the $\frac{1}{24}$ grain strychnia sulphate. The tr. ferri muriat is sometimes used.

*Rupia.*

A young man was under treatment, receiving of the potassium iodide eight grains, with two grains of the muriate of ammonia; the dose repeated three times daily; topically, a lotion of carbolic acid. The eruption in this patient is due to a venereal ulcer contracted a year ago, has widely spread over all parts of the body, and is a typical and fine illustration of rupia.

In the treatment of syphilis, care is taken to bring the condition of the patient up to a good standard by nutritious diet, and by the syrup iodide of iron with bark, before the usual specific treatment is ordered.

A young girl of sixteen had an attack of scarlatina in her childhood, which, among other evils, produced deafness. She had never menstruated, but an oval globular tumor above the pubis had been gradually forming and enlarging. Upon dilating and incising the cervix uteri, the contents of this tumor were evacuated, and consisted of retained menstrual fluid and detritus. A few weeks later, imprudent exposure and over-exercise brought on an attack of cellulitis and peritonitis, with formation of abscess in the left inguinal region. The patient's condition was one of great debility, pain upon moving, or on being touched over the region of the abscess. After waiting for some return of strength, the fluid was drawn off by the aspirator, its puncture being made in the left iliac region, on a line with the centre of Poupart's ligament, and four inches above the ligament.

Subsequently, a spontaneous opening formed two inches above the point of the first puncture, possibly because the abscess may have been divided into two cysts or compartments, into one of which the needle of the aspirator did not penetrate.

*Excessive Tympanites.*

A patient convalescing from typhoid fever, exhibited a most wonderful gaseous distention of the abdomen. To relieve this, bismuth, turpentine and various other medicines and clysters of many sorts were ordered, but without benefit. Upon the physiological grounds of its well-known action upon the unstriped muscular fibres, the fluid extract of ergot, twenty minims four times a day, was given, with speedy and permanent effect upon the intestine; the tympanites soon disappeared.

*Delirium Tremens.*

Dr. Howell gives the fluid extract of cannabis indica where the patient is in a very nervous and tremulous condition (where he can neither sit nor stand), twenty or thirty minims in one dose at night, usually without the potassium bromide. The hydrate of chloral has been observed to cause distress to the eyes, and, in one case, a conjunctivitis has been thought to have followed the use of chloral.

Formula to disguise the taste of cod-liver oil: Glycerine, two parts; brandy, one part, and cod-liver oil, one part. Mix, and add enough of essence of wintergreen to flavor.

July 31. Surgical Clinic, by Dr. FREER.

*Reunited Fracture of the Left Leg.*

This man broke his leg six months ago. The line of separation is at the junction of the upper and middle third of the limb; it is only partially united, probably because the surfaces of the ends of the fragments are covered with fibrous, ligamentous structure, thus preventing the formation of bone structure.

The treatment for such a non-union will vary with the interval after time of accident. The moderate means, such as the movement of the ends upon each other, blistering, or walking on the limb, should first be tried. Too long a time has elapsed to allow any expectations of such means being of benefit in this case.

I shall employ a more radical method, one devised and introduced in 1850 by the late Prof. Brainard—the boring of the bone by a drill. It will succeed in all proper cases for that kind of treatment. No good results are to be expected when the drill is used in patients very cachectic, anæmic, who have cancer, or are in gestation or lactation. Its design is to denude the surface of the bone, excite moderate inflammation and *reproduction* of bone; the drill acts by displacement. Very little bone dust or chips are formed. The seton and the ivory peg do not cause reproduction of bone, though they set up an inflammation.

In his experiments upon the lower animals, Dr. Brainard found that the ivory peg, driven into the seat of fracture, would cause necrosis, or at least destruction of bone.

The integument should be moved above or below the point where you intend to insert the drill, in order that when the skin falls to its natural position, it may cover over the points you have drilled in the bone. Pass the drill through the superficial to the deeper fragments of bone ; then, without entirely withdrawing the drill, move it to one or the other side, drawing the skin with it. Each fragment, the distal and proximal, may in this way be drilled in three or four places.

It may be necessary to repeat this operation, at intervals of a few weeks, three or four times.

Dr. Freer made three openings in each fragment, the drill going through the entire diameter of the bone. No anæsthesia was needed. The leg will be put at once into a plaster of Paris splint.

